# Emergency versus Elective Cervical Cerclage: An Audit of Our First Two Years of Service

**DOI:** 10.1155/2018/2065232

**Published:** 2018-09-30

**Authors:** N. Vasudeva, C. Reddington, M. Bogdanska, L. De Luca

**Affiliations:** Obstetrics and Gynaecology Department, Sunshine Hospital, Melbourne, Australia

## Abstract

One of the biggest obstetric challenges is the diagnosis and management of a short cervix as cervical length has an inverse relationship with risk of preterm birth. A cervical cerclage is a surgical procedure to reduce the risk of preterm birth and can be placed in an elective or emergency setting. This is a retrospective review of cervical cerclages inserted at an outer metropolitan hospital from February 2014 to May 2017. Since the introduction of the service, a total of 43 patients were identified as requiring a cervical cerclage. Four of these patients were transferred to tertiary hospitals. Of the 39 cerclages inserted, 26 were elective and 13 were emergency, placed at a mean gestation of 15.6 and 19.6 weeks. In total, there were 35 live births, 2 stillbirths, and 2 neonatal deaths. The maternal demographics (age, gravidity, parity, and preterm risk factors) were not statistically significant between the two groups. The mean pregnancy prolongation and birthweight was greater in the elective than the emergency group (21.4 versus 14.1 weeks; 3148.2 versus 2447.2 grams). There was no obvious pattern with which patients received antibiotics pre-, intra-, or postoperatively or received a vaginal swab. This audit identified the need for improvements to guidelines to standardise the use of antibiotics and progesterone in women with a cervical cerclage.

## 1. Introduction

Cervical insufficiency, earlier known as cervical incompetence, is the process of painless cervical dilatation, resulting in second trimester pregnancy loss in an otherwise normal pregnancy [1, 2]. It is thought to occur in 1% of the obstetric population and suspicion is raised when there is a history of recurrent midtrimester loss, previous preterm delivery, or a previous short cervix [2]. One of the biggest obstetric challenges is the diagnosis and management of a short cervix as multiple different definitions and guidelines exist [3].

Cervical cerclage is an obstetric procedure first described in the 1950s, where a suture is placed around the cervix for prevention of preterm birth (PTB) [3]. PTB is defined as delivery prior to 37 weeks of gestations and can lead to increase morbidity and mortality [3–6]. The World Health Organisation defines PTB <28 weeks as extreme preterm, 28-32 weeks as very preterm, and 32-37 weeks as late preterm [7].

The cervical length is most accurately measured using transvaginal ultrasound as it is more reproducible than transabdominal ultrasound [6]. The measurement will vary depending on the gestation, with shortening occurring as the pregnancy continues. At 22-25 weeks of gestation, the median cervical length is expected to be 35mm. The significance of a short cervix is the inverse relationship it has with risk of PTB; the shorter the cervix, the higher the risk of PTB. The risk is increased if other preterm risk factors are present [8].

A short cervix is defined as one which is less than the 10th centile (25mm) in the midtrimester pregnancy [5, 8, 9]. Studies have shown that interventions at this cervical length improve pregnancy outcomes in both low and high-risk women [9]. The management of patients with short cervix can include surveillance, progesterone pessaries, or insertion of a cervical cerclage and there is evidence that such interventions may reduce the risk of PTB [3, 5, 10, 11].

A cervical cerclage is frequently categorised as an elective or emergency cerclage [4, 9]. An elective cerclage is usually placed at the end of the first trimester. The indication for an elective cerclage is based on prior obstetric history. An emergency cerclage can be placed up to 24 weeks of gestation and is indicated when there is a visibly dilated cervix on speculum examination or if there has been an unexpected finding of a shortened cervix on routine ultrasound examination [4, 10–12]. Trials looking at outcomes between emergency and elective cerclage have shown that pregnancy outcomes are comparable, although some have also shown poorer obstetric outcomes in the emergency cohort [12, 13].

Sunshine Hospital is an outer Melbourne secondary hospital. A cervical surveillance clinic for the prevention of PTB and midtrimester miscarriage was established in 2014. A guideline for the management of a short cervix in this institution was implemented in 2015. In order to review the outcomes of all cervical cerclages placed during in this period, a retrospective audit of all cerclages placed from 2014 to 2017 was conducted.

## 2. Method

Data of all singleton pregnant women who underwent an elective or emergency cervical cerclage between February 2014 and May 2017 at a single secondary obstetric centre were reviewed. Ethics approval was provided by the Ethics Committee at Western Health. No funding was received for this study. Patient data were collated from the hospital's electronic systems, Birthing Outcome Systems (BOS) and BOSSNET. The data was collected onto a Microsoft Excel spread sheet. A statistical online package was used for the Pearson Chi-squared test analysis.

In the elective cerclage group, the need for a cerclage was determined by past risk factors. These risk factors included previous PTB below 30 weeks' gestation, midtrimester loss, cervical surgery, cervical trauma, or congenital uterine malformations. In the emergency cerclage group, the need for a cerclage was identified by an unexpected ultrasound finding of a shortened cervix (less than the 10th centile in midtrimester) or clinically from a speculum examination (dilated cervix with or without bulging membranes).

Patient demographics collected included age, gravidity, parity, and body mass index (BMI) as well as any cervical insufficiency risk factors. The indication for cervical cerclage and other factors related to the cerclage insertion were collected including cervical length and/or dilatation, presence of ‘sludge', type of cerclage, and suture material used. Delivery outcomes reviewed included gestation at delivery, birthweight, neonatal survival, and the presence of preterm prelabour rupture of membranes (PPROM) or chorioamnionitis. The use of antibiotics in the group of women receiving a cerclage was reviewed to determine if a standardised approach to prescribing existed and if there was adherence to the institutional guideline.

## 3. Results

All patients underwent a McDonald Cerclage under general anaesthesia with the majority using Mersilene tape as the preferred suture material. All cerclages were placed by a senior registrar (under consultant Obstetrician supervision) or by a consultant Obstetrician.

**Figure 1:**
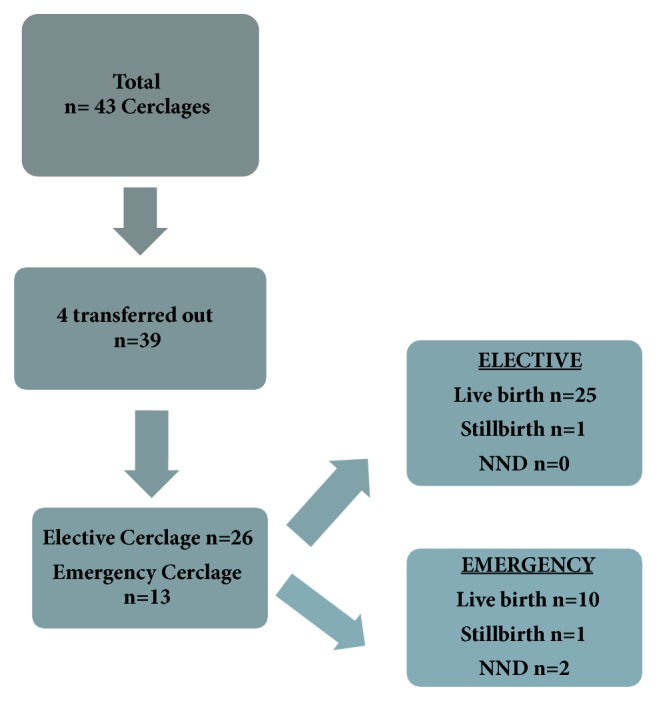
Pregnancy outcomes.

In total, there were 43 women identified as requiring a cervical cerclage. Three women were transferred to a tertiary centre due to the unavailability of a suitably trained surgeon. Of the 39 cerclages inserted at our service, 26 were elective and 13 were emergency cerclages. In the elective cerclage group, there were 25 live births and 1 stillbirth. In the emergency cerclage group, there were 10 live births, 1 stillbirth, and 2 neonatal deaths (NND) (Figure 1).

Maternal demographics and risk factors are summarised in Table 1. The mean age, gravidity, parity, and BMI was similar in the two groups. The number of risk factors for cervical insufficiency was increased in the elective cerclage group.

The mean gestational age at cerclage insertion was 15+6 weeks in the elective group compared with 19+6 weeks in the emergency group. Women in the elective group had a mean cervical length of 27.2mm with no cervical dilatation whilst women in the emergency group had a mean cervical length of 14.6mm and a mean 16mm dilated cervix. There was a statistically significantly higher rate of ‘sludge' present in the emergency cerclage cohort (7.6 versus 61.5% p<0.00068). Following placement of an emergency cerclage, there was greater use of antibiotics and vaginal progesterone. (23.1 versus 69.3% p<0.0126 and 57.7 versus 92.3% p<0.033) (Tables 2 and 3).

Women were more likely to deliver at a later gestation in the elective group with a mean gestation of 37 weeks compared with 34 weeks in the emergency cerclage group. The mean pregnancy prolongation and birthweights were greater in the elective cerclage group. An increased rate of preterm prelabour rupture of membranes (PPROM) was observed in the emergency cerclage group with 75% occurring in the extreme preterm period. The rate of extreme preterm PPROM was 16.7% in the elective cerclage group. There were no identified cases of chorioamnionitis in either group (Table 4).

## 4. Discussion

This retrospective review found that elective placement of cervical cerclage had a trend towards better pregnancy outcomes as has been previously established. A greater pregnancy prolongation period was observed with an expected greater mean birthweight compared with the emergency cerclage group. The rates of neonatal death and stillbirths was reduced in the elective cerclage group. Pregnancy prolongation decreases PTB, which improves neonatal morbidity and mortality and also reduces the economic burden on the healthcare system. The recent Western Australia Preterm Birth Prevention Initiative has been working towards such goals [11].

The placement of an emergency cerclage conferred benefits with a mean pregnancy prolongation of 14+1 weeks, improving neonatal survival. The liveborn rate was 76.1% with a mean gestation of 33.7 weeks at delivery. There was an increased rate of PPROM in the emergency group (23% versus 30.8%) but no other cerclage associated complications. It is known that the risk of complications can be higher in the setting of an emergency cerclage [12].

In the emergency group, the two NND and the one stillbirth were all in women with pregnancies that resulted in PPROM at an extreme preterm gestation. In the elective cerclage group, the only stillbirth was also following PPROM at an extreme preterm gestation. This finding is similar to previous studies which have demonstrated that emergency cerclage has a higher risk of poorer obstetric outcomes [13].

There was also a statistically higher rate of ‘sludge' present in the emergency cerclage cohort (7.6 versus 61.5% p<0.00068). Sludge is intra-amniotic debris which is in close proximity to the internal cervical os. It is thought to be a marker for subclinical intra-amniotic infection, but this remains contentious [14]. A recent study by Adanir et al. demonstrated that sludge is an independent risk factor for PTB [14].

In this audit, there were significant inconsistencies with the use of antibiotics pre-, intra-, and postoperatively in both type of antibiotic used and length of use in our cohort. There are no trials that support the use of antibiotics with cerclage; however broad-spectrum antibiotics are commonly prescribed intraoperatively [13]. The institutional guideline gave no guidance for the use of antibiotics with cervical cerclage.

The guideline does recommend performing vaginal swabs in the presence of a short cervix and treatment for organisms known to potentially contribute to preterm labour but is not explicit in its recommendation pre-cerclage. Furthermore, there is no evidence to support the routine use of progesterone with cervical cerclage; however in our cohort, progesterone was used frequently after insertion, particularly in the emergency cerclage group [5].

The limitations to this study are the small number of cases identified which can limit the generalisability of this study and the retrospective nature of this review.

## 5. Conclusion

This audit has identified with the introduction of a cervical surveillance clinic at Sunshine Hospital that women at risk of midtrimester miscarriage and PTB are being identified and management instituted. The outcomes for both elective and emergency cerclage are good with low rates of complications documented. There are inconsistencies with some aspects of care in women receiving cervical cerclages with deficiencies in the institutional guideline available for clinicians. This audit supports the need for a cervical surveillance clinic and an improved evidence based guideline. The will aid in standardising the care for at risk women for better pregnancy outcomes.

## Figures and Tables

**Table 1:** Maternal demographics.

	**All Cerclage ** **(n=39)**	**Elective Cerclage ** **(n=26)**	**Emergency Cerclage ** **(n=13)**	**P-Value ** **(Elective vs Emergency) **
**Mean maternal age**	31.3	31.7	30.6	

**Mean gravidity **	4.0	4.0	3.7	

**Mean parity **	1.7	1.7	2.0	

**Mean BMI (kg/m** ^**2**^ **)**	27.3	27.0	27.8	

**Risk Factors (**%**) **				
**MTL **	27 (69.2)	20 (76.9)	7 (53.8)	0.16
**PTL**	7 (17.9)	7 (26.9)	0 (0)	0.07
**Cervical surgery **	9 (23.1)	6(23.1)	3 (23.1)	1.0
**Uterine**	2 (5.1)	2 (7.7)	0 (0)	0.543

*MTL: midtrimester loss.*

*PTL: preterm loss.*

*Uterine: uterine congenital malformations.*

**Table 2:** Cervical results.

	**Total Cerclage ** **(n=39)**	**Elective Cerclage ** **(n=26)**	**Emergency Cerclage ** **(n=13)**	**P-Value ** **(Elective vs Emergency) **
**Mean cervical length (mm)** **∗**	23.0	27.2	14.6	

**Mean cervical dilation (mm)+**	16.0	0	16.0	

**Presence of sludge (**%**) **	10 (25.6)	2 (7.6)	8 (61.5)	0.000688*∗∗*

**Mersilene Suture Material (**%**) **	27 (69.2)	18 (69.2)	9 (69.3)	1.0

**Cervical Cultures (**%**)**				
**Candida**	8 (20.5)	6 (23.1)	2 (15.4)	0.694
**Ureaplasma **	2 (5.1)	1 (3.8)	1 (7.7)	1.0
** BV**	1 (2.6)	1 (3.8)	0 (0)	1.0

*∗Total n= 31, Elective n= 21, and Emergency n=10.*

*Others excluded as length not specified in report.*

*+Total n=6; Emergency n=6.*

*Others excluded as dilation not present or not specified in report.*

*∗∗ Statistically significant.*

**Table 3:** Antibiotic and progesterone use.

	**Total Cerclage ** **(n=39)**	**Elective Cerclage ** **(n=26)**	**Emergency Cerclage ** **(n=13)**	**P-Value ** **(Elective vs Emergency) **
**Antibiotic Pre-Op (**%**) **	3 (7.7)	1 (3.9)	2 (15.4)	0.253

**Antibiotic Intra-Op (**%**)**	28 (71.8)	18 (69.2)	10 (76.9)	0.719

**Antibiotic Post-Op (**%**)**	15 (38.5)	6 (23.1)	9 (69.3)	0.0126*∗*

**Progesterone Pre-Op (**%**)**	12 (20.8)	6 (23.1)	6 (46.2)	0.163

**Progesterone Post-op (**%**)**	27 (69.2)	15 (57.7)	12 (92.3)	0.033*∗*

*∗*Statistically significant.

**Table 4:** Obstetric and pregnancy outcomes.

	**Total Cerclage** **(n=39)**	**Elective Cerclage ** **(n=26)**	**Emergency Cerclage ** **(n=13)**
**Mean GA cerclage placed (weeks) **	16.9	15.6	19.6

**Mean GA at delivery (weeks) **	35.9	37.0	34

**Mean Pregnancy Prolongation (weeks) **	19.0	21.4	14.1

**Mean Birthweight (grams) **	2914.6	3148.2	2447.2

**Chorioamnionitis (**%**) **	0 (0)	0 (0)	0 (0)

**PPROM (**%**) **	10 (24.6)	6 (23.0) *(i) 1(16.7): extreme preterm* *(ii) 1 (16.7): very preterm* *(iii) 4 (66.7): late preterm*	4 (30.8) *(i) 3 (75): extreme preterm* *(ii) 1 (25): late preterm *

**Liveborn (**%**) **	35 (94.5)	25 (96.2)	10 (76.2)

**NND (**%**) **	2 (5.1)	0 (0)	2 (15.4)

**Stillborn (**%**) **	2 (4.1)	1 (3.8)	1 (7.7)

*GA: gestational age.*

*PPROM: premature prelabour rupture of membranes.*

*NND: neonatal death.*

## Data Availability

This information can be provided on request. The data collected is on a password protected excel file.
